# Large-scale association analysis of *TNF*/*LTA *gene region polymorphisms in type 2 diabetes

**DOI:** 10.1186/1471-2350-11-69

**Published:** 2010-05-06

**Authors:** Vesna Boraska, Nigel W Rayner, Christopher J Groves, Timothy M Frayling, Mahamadou Diakite, Kirk A Rockett, Dominic P Kwiatkowski, Aaron G Day-Williams, Mark I McCarthy, Eleftheria Zeggini

**Affiliations:** 1 Department of Medical Biology, University of Split School of Medicine, Split, Croatia; 2 Wellcome Trust Centre for Human Genetics, University of Oxford, Oxford, UK; 3 Oxford Centre for Diabetes, Endocrinology and Metabolism, University of Oxford, Oxford, UK; 4 Peninsula Medical School, University of Exeter, Exeter, UK; 5 Wellcome Trust Sanger Institute, Wellcome Trust Genome Campus, Hinxton, Cambridge, UK

## Abstract

**Background:**

The *TNF*/*LTA *locus has been a long-standing T2D candidate gene. Several studies have examined association of *TNF*/*LTA *SNPs with T2D but the majority have been small-scale and produced no convincing evidence of association. The purpose of this study is to examine T2D association of tag SNPs in the *TNF*/*LTA *region capturing the majority of common variation in a large-scale sample set of UK/Irish origin.

**Methods:**

This study comprised a case-control (1520 cases and 2570 control samples) and a family-based component (423 parent-offspring trios). Eleven tag SNPs (rs928815, rs909253, rs746868, rs1041981 (T60N), rs1800750, rs1800629 (G-308A), rs361525 (G-238A), rs3093662, rs3093664, rs3093665, and rs3093668) were selected across the *TNF*/*LTA *locus and genotyped using a fluorescence-based competitive allele specific assay. Quality control of the obtained genotypes was performed prior to single- and multi-point association analyses under the additive model.

**Results:**

We did not find any consistent SNP associations with T2D in the case-control or family-based datasets.

**Conclusions:**

The present study, designed to analyse a set of tag SNPs specifically selected to capture the majority of common variation in the *TNF*/*LTA *gene region, found no robust evidence for association with T2D. To investigate the presence of smaller effects of *TNF*/*LTA *gene variation with T2D, a large-scale meta-analysis will be required.

## Background

Type 2 diabetes (T2D) is a complex disease influenced by environmental and genetic factors. Genetic association studies have thus far identified at least 20 replicating T2D susceptibility loci of modest to small effect, which together explain less than 10% of the genetic component of disease [1,2]. Several genome-wide association scans (GWAS) have been carried out for T2D [3-10]. These have used a variety of genotyping platforms with different SNP content, typically capturing over 80% of common variation in European-descent populations. Although this extent of coverage, in combination with imputation approaches [11], reduces the need for candidate gene studies, in-depth investigation of variation at loci of interest can conceivably prove useful in characterising them further.

The *TNF*/*LTA *locus has been a long-standing T2D candidate gene. T2D and obesity have been hypothesised to have an inflammatory basis [12,13]. Insulin resistance is associated with increased plasma levels of proinflammatory cytokines such as TNF and IL6, and with interactions between TNF and NFkappaB that lead to an increase of oxidative stress [14-16].

The genes coding for TNF and LTA reside in the class III MHC region on chromosome 6p21.3. TNF and LTA are members of the TNF ligand superfamily, bind the same TNF receptors and mediate similar pleiotropic effects [17,18]. Of the multiple SNPs in the *TNF*/*LTA *gene region, the rs361525 (G-238A) and rs1800629 (G-308A) *TNF *promoter variants, and the rs1041981 (T60N) *LTA *variant have been the most frequently studied in T2D. The majority of studies of *TNF*/*LTA *SNPs have been small-scale, with some notable exceptions [17], and have produced no convincing evidence for association with the disease [19-27].

The Wellcome Trust Case Control Consortium (WTCCC) T2D GWAS examined 17 directly typed and imputed SNPs from the *TNF*/*LTA *gene region and detected no association with T2D in 2000 cases and 3000 controls from the UK [6,28]. In addition, a GWAS meta-analysis for T2D carried out by the DIAGRAM consortium, which examined the same 17 directly genotyped and imputed SNPs in the *TNF/LTA *region in samples from three sources (Diabetes Genetics Initiative (DGI), Finland-United States Investigation of NIDDM Genetics (FUSION) and WTCCC) also found no association between *TNF*/*LTA *SNPs and T2D [28]. However, the WTCCC genotyping platform (Affymetrix 500k) and HapMap-based imputation do not provide exhaustive coverage of common variation in this gene region. To increase coverage, we carried out a genetic association study of the *TNF*/*LTA *loci in a total of 5359 samples from the UK by typing additional SNPs, selected on the basis of sequence data to better capture variation in the region.

## Methods

### Subjects

This study comprised a case-control and a family-based component. The case-control dataset included 1520 cases from the Diabetes UK Warren 2 Sib Pair Repository (61.5% males) and 2570 control samples from the 1958 British birth cohort (n = 2027, 50.6% males) [29], and the HRC control collection (n = 543, 49.9% males) derived from UK blood donors and available from the European Centre for Cell Culture (ECACC, CAMR, Salisbury, UK). The family-based dataset comprised 423 parent-offspring trios (58.5% male probands) from the Diabetes UK Warren 2 Trios (W2T) Repository. W2T probands were selected by strict clinical, immunological and genetic criteria as previously described [30]. All cases included in the present study had T2D diagnosed according to the World Health Organization criteria and were selected for early diabetes onset and/or positive family history. Importantly, autoimmune diabetes was excluded based on GAD antibody typing, age of disease onset above 25, insulin independence following diagnosis, no ketoacedosis and no first degree relatives with type 1 diabetes [30,31]. Clinical characteristics of the cases are provided in Table 1. 58.2% of WTCCC cases and 35.3% of WTCCC controls overlapped with the samples examined as part of our study. All subjects were exclusively of UK/Irish origin and provided signed informed consent prior to blood sampling. Reported investigations have been carried out following the principles of the Declaration of Helsinki as revised in 2000. Ethical oversight for collection and use of the T2D cases was provided from MREC 00/6/55, Peterborough and Fenland LREC 05/Q0106/78 and from over 100 individual local research ethics committee approvals. Use of the 1958 Birth Cohort samples is in accordance with Joint UCL/UCLH Research Ethics Committee A approval 08/H0714/40 and South-East Multi-Centre Research Ethics Committee approval MREC 01/1/44. The HRC samples are a commercially available set of anonymised DNA samples from blood donors sourced from the Health Protection Agency Culture Collection and approved for research use only.

**Table 1:** Clinical characteristics of T2D cases

Cohort	Female	Male	Average Age At Study (years)	Average AOD^c ^(years)	Average BMI (kg/m^2^)
W2C^a^	583	932	60.23	51.46	31.80

W2T^b^	173	249	46.26	40.44	32.91

^a^W2C - Warren 2 Cohort; ^b^W2T - Warren 2 Trios probands; ^c^AOD - Age of diagnosis

### SNP Selection and Genotyping

Eleven haplotype-tagging SNPs (tag SNPs) (rs928815, rs909253, rs746868, rs1041981 (T60N), rs1800750, rs1800629 (G-308A), rs361525 (G-238A), rs3093662, rs3093664, rs3093665, rs3093668) were selected across the *TNF *and *LTA *loci from sequencing and genotype data generated in 32 Caucasian trios as previously described [32,33] (Table 2). Genotypes for this study were determined using a fluorescence-based competitive allele specific assay (Kaspar, Kbioscience, UK). Genotyping and SNP selection details are available from the authors on request. When we investigated linkage disequilibrium (LD) between the SNPs, we found SNPs rs928815 and rs746868, and SNPs rs909253 and rs1041981 to be in tight LD with each other (r^2 ^> 0.8). SNP pairs rs361525-rs3093662, rs361525-rs3093668 and rs3093662-rs3093664 displayed moderate LD (0.6 < r^2 ^< 0.8), while all other investigated SNPs showed no or very weak pairwise LD (0 < r^2 ^< 0.52) (Additional file 1, Figures S1a and S1b). Eight of the 11 studied tag SNPs have been genotyped in the HapMap (rs928815, rs909253, rs1041981, rs1800750, rs1800629, rs3093662, rs3093665, rs3093668). These 8 variants capture 61.1% of common (MAF > 0.05) variation across the *TNF*/*LTA *gene region (overall 18 SNPs in the HapMap database) at an r^2 ^threshold of ≥0.8, based on the HapMap (CEU population, Rel 24/PhaseII Nov 08 [34]). The proportion of common variation across the region captured by the typed tag SNPs was calculated on a multimarker tagging basis using Tagger [35]. However, this is an underestimate of the capture of common variation, since the 3 remaining non-HapMap variants were excluded from this calculation. To asses coverage further we performed another calculation on the basis of the 1000 genomes project data (http://www.1000genomes.org) April 2009 release).

**Table 2:** Characteristics of 11 *TNF*/*LTA *tag SNPs.

dbSNP rs number	Position on chr. 6^a^	Alternative name	Gene location	Alleles (C:M)^b^	dbSNP CEU MAF^c^
rs928815	31639194	/	5' *LTA*	G:T	0.383

rs909253	31648292	252G > A	*LTA*-intron 1	T:C	0.358

rs746868	31648408	/	*LTA*-intron 1	G:C	0.405

rs1041981	31648763	Thr26Asn;T60N; 804C > A	*LTA*-exon 3	C:A	0.358

rs1800750	31650942	-376 G > A	5' of *TNF*	G:A	0.008

rs1800629	31651010	-308 G > A	5' of *TNF*	A:G	0.217

rs361525	31651080	-238 G > A	5' of *TNF*	G:A	0.068

rs3093662	31652168	IVS1-122A > G; +851	*TNF*-intron 1	A:G	0.071

rs3093664	31652621	/	*TNF*-intron 3	A:G	0.065

rs3093665	31653370	/	*TNF *- 3'UTR	A:C	0.017

rs3093668	31654474	/	3' of *TNF*	G:C	0.042

^a^Based on UCSC Genome Browser; ^b^C:M - common allele: minor allele; ^c^MAF - minor allele frequency for the CEU population based on NCBI dbSNP database

### Statistical analysis

Quality control (QC) of the obtained genotypes was performed prior to association analysis. The SNP genotyping success rates ranged from 93.3% to 98.6%. We evaluated the comparative rate of missing genotypes between cases and controls using Plink (version 1.00) [36] and excluded rs3093662 from the case-control association analysis due to low call rate. The tag SNPs were tested for deviation from Hardy-Weinberg equilibrium (HWE) in affected and healthy individuals separately using Stata v. 8 (Stata Corporation, College Station, TX, USA) and Plink (version 1.00) [36]. No deviations from HWE were observed. Minor allele frequencies (MAFs) of controls in both studies were compared with the National Center for Biotechnology Information SNP database (NCBI dbSNP) MAFs for the CEU population and showed no significant differences. Testing of Mendelian inheritance using Plink and Haploview [36,37] identified inconsistencies in one family, which was excluded from further analysis. After QC, 10 tag SNPs were taken forward to case-control association analyses and 11 tag SNPs were included in family-based association analysis.

Single-point case-control association analyses were carried out using Stata v. 8 (Stata Corporation, College Station, TX, USA). Multi-point case-control association analyses of fixed haplotype sizes (sliding windows of 2-10 SNPs shifting 1 SNP at a time) were performed using the expectation-maximisation algorithm-based approach implemented in Plink [36]. Single-point and multi-point (sliding windows of 2-11 SNPs) family-based association analyses were carried out using implementations of the transmission disequilibrium test (TDT) in Plink [36]. 10,000 permutations were run for each association analysis. r^2 ^and D' measures of pairwise LD were calculated for all SNPs using Haploview [37]. Power was calculated under the log-additive model for a range of effect-sizes (1.1<OR>2) at α = 0.05 using Quanto [38]. All association analyses are unadjusted (e.g. for BMI, blood pressure and other environmental variables), as these data were not available to us. We did not investigate gene-environment interactions.

## Results

Genotype distributions for the 11 *TNF*/*LTA *tag SNPs in the case-control and parent-offspring datasets are shown in Additional file 1, Table S1 and Table S2, respectively. Overall, we did not identify any consistent significant SNP associations with disease. The most frequently studied SNPs, rs1800629 (G-308A), rs361525 (G-238A), and rs1041981 (T60N) did not show robust association with the disease in any dataset (Table 3). Exhaustive multimarker case-control analyses did not identify any strong haplotypic associations (data not shown). There were no statistically significant deviations in the transmission of alleles from parents to affected probands by single-point (Table 4) or haplotype-based analysis (data not shown). Our study had 80% power at α = 0.05 to identify modest/large effect sizes (OR > 1.3) at common loci (Additional file 1, Tables S3a and S3b).

**Table 3:** Case-control association analysis results for the 10 *TNF*/*LTA *tag SNPs

SNP	OR allele	**OR**^**a**^	**95% CI**^**a**^	p value
rs928815	T	1.056	0.96-1.16	0.255

rs909253	T	0.889	0.81-0.98	0.015

rs746868	C	1.066	0.97-1.17	0.18

rs1041981 (T60N)	C	0.892	0.81-0.98	0.019

rs1800750	A	1.001	0.64-1.54	0.996

rs1800629 (G-308A)	A	0.984	0.88-1.11	0.791

rs361525 (G-238A)	G	0.981	0.81-1.19	0.845

rs3093664	A	0.996	0.85-1.17	0.958

rs3093665	C	1.192	0.86-1.64	0.268

rs3093668	C	1.053	0.85-1.3	0.625

^a^allelic OR and 95% confidence intervals.

**Table 4:** Transmission disequilibrium analysis of 11 *TNF*/*LTA *tag SNPs in T2D parent-offspring trios

SNP	**N**^**a**^	**A1:A2**^**b**^	**MAF**^**c**^	**T:U**^**d**^	**OR**^**e**^	95% CI ^f^	p value
rs928815	322	T:G	0.359	132:165	0.8	0.64-1.01	0.055

rs909253	321	C:T	0.358	157:145	1.083	0.86-1.36	0.489

rs746868	324	C:G	0.362	135:163	0.828	0.66-1.04	0.104

rs1041981 (T60N)	301	A:C	0.355	146:138	1.058	0.84-1.34	0.635

rs1800750	327	A:G	0.012	7:12	0.583	0.23-1.48	0.251

rs1800629 (G-308A)	237	G:A	0.195	73:70	1.043	0.75-1.45	0.801

rs361525 (G-238A)	324	A:G	0.055	35:35	1	0.63-1.59	1

rs3093662	321	G:A	0.079	47:44	1.068	0.71-1.61	0.753

rs3093664	321	G:A	0.086	55:43	1.279	0.86-1.91	0.225

rs3093665	324	C:A	0.023	14:12	1.167	0.54-2.52	0.694

rs3093668	321	C:G	0.045	31:22	1.409	0.82-2.43	0.216

^a^N - number of informative trios; ^b^A1:A2 - minor allele *vs*. major allele; ^c^MAF - minor allele frequency in affected probands; ^d^copies of the minor allele transmitted (T) and untransmitted (U), ^e^odds ratios (OR), ^f^95% lower and upper confidence intervals.

In the WTCCC GWAS [5,28], a total number of 17 (one directly genotyped, rs1799964, and 16 imputed) SNPs from the *TNF*/*LTA *gene region were investigated and showed no association with T2D. The case-control association results of the most frequently studied SNPs, rs1800629 (G-308A), rs361525 (G-238A) and rs1041981 (T60N) from the present study and from the WTCCC dataset (across which there is considerable overlap) is shown in Table 5. Five of the tag SNPs from the present study (rs746868, rs1800750, rs361525, rs3093664 and rs3093665) were not directly typed or imputed in the WTCCC GWAS. To assess the extent of additional coverage these 5 SNPs offer, we examined LD based on our genotype data in T2D cases and controls. SNPs rs746868 and rs361525 are in high LD (r^2 ^= 0.99 and r^2 ^= 0.79) with two of the WTCCC-typed SNPs, rs928815 and rs3093668 respectively. The remaining 3 SNPs that have not been examined in the WTCCC (rs1800750, rs3093664 and rs3093665) demonstrate low LD with WTCCC-typed and other HapMap SNPs (0 < r^2 ^< 0.52) (Additional file 1, Figures S1a and S1b). Therefore these polymorphisms capture additional variation missed by the WTCCC study.

**Table 5:** The comparison of association results for T60N, G-308A and G-238A between the present study and the WTCCC T2D GWAS

SNP	OR allele	**OR**^**a**^	**95% CI**^**a**^	p-value	**WTCCC SNP**^**b**^	**OR**^**a**^	**95% CI**^**a**^	p-value
rs1041981 (T60N)	C	0.89	0.81-0.98	0.02	rs1041981	1.06	0.96-1.16	0.67

rs1800629 (G-308A)	A	0.98	0.88-1.11	0.79	rs1800629	1.05	0.93-1.19	0.56

rs361525 (G-238A)	G	0.98	0.81-1.19	0.85	rs3093668 (proxy for rs361525)	0.86	0.71-1.05	0.21

^a^OR - allelic odds ratio and 95% confidence intervals; ^b^All three SNPs were imputed in the WTCCC data set. 58.2% of WTCCC cases and 35.3% of WTCCC controls overlapped with the samples from our study.

We investigated capture further on the basis of the 1000 genomes project data. Four of our 11 tag SNPs (rs909253, rs1800750, rs3093662 and rs3093665) were not found in the 1000G dataset and the remaining 7 tag SNPs capture 60.6% of common variation (overall 33 *TNF*/*LTA *SNPs in the 1000G dataset) on a multimarker tagging basis at an r^2 ^threshold of ≥0.8. This is again an underestimate of the *TNF*/*LTA *common variation capture by our tag SNPs.

## Discussion

In this study of 11 tag SNPs, we find no consistent evidence for association between *TNF*/*LTA *region variation and T2D. The present study was designed to analyse a set of tag SNPs specifically selected to capture the majority of common variation in the *TNF*/*LTA *gene region based on proprietary sequence and genotype data [32,33]. Although a proportion of the investigated variants had been examined as part of the WTCCC GWAS [5,28], this study provides further capture of common variation across the region. However, the overall conclusion remains unchanged - there was no evidence of association with disease.

This is one of the largest studies to date, showing no association between *TNF/LTA *variation and T2D. A recent meta-analysis (2106 cases and 2920 controls) of the rs361525 (G-238A) variant did not detect a significant association with T2D [23]. Similar meta-analyses of all reported association studies for the rs1800629 (G-308A) and rs1041981 (T60N) SNPs, which have been widely investigated with respect to T2D, may boost power to detect possible small effects at these loci.

T2D is a complex disease caused by complex interplay between environmental and genetic factors. A limitation of our study is that we have not been able to adjust for or investigate interaction of SNPs with BMI, age, gender, blood pressure, serum lipid levels etc. as these data were unavailable to us. In addition, even though our study examined the majority of common variation across the region, it is possible that causal, associated variants may have been missed.

## Conclusions

The purpose of this study was to examine if genetic variation in the genes encoding inflammatory proteins TNF and LTA alter the risk of developing T2D. We tested a carefully selected set of haplotype tagging SNPs that capture the majority of common variation in the *TNF*/*LTA *gene region in case-control and parent-offspring samples and find no robust evidence for association. Large-scale meta-analyses will be required to investigate the presence of smaller effects at polymorphic sites in the *TNF*/*LTA *gene region.

## Abbreviations

CEU: central European population; GWAS: genome wide association study; HWE: Hardy-Weinberg equilibrium; LD: linkage disequilibrium; LTA: lymphotoxin alpha; MAF: minor allele frequency; QC: quality control; SNP: single nucleotide polymorphism; tag SNP: tagging SNP; TDT: transmission disequilibrium test; TNF: tumor necrosis factor; T2D: type 2 diabetes mellitus.

## Competing interests

The authors declare that they have no competing interests.

## Authors' contributions

VB carried out statistical analysis, participated in interpretation of results and drafted the manuscript. NWR managed the data files and participated in statistical analyses. CJG directed the genotyping and managed the samples. TMF participated in study design. MD, KAR and DPK contributed sequence data for the *TNF*/*LTA *gene region and to manuscript preparation. AGDW contributed to study design, analysis and manuscript preparation. MIM conceived and coordinated the study, participated in study design, interpretation and revised the manuscript. EZ supervised the study and participated in study design, statistical analysis, interpretation, draft and revision of the manuscript. All authors read and approved the final version of the manuscript.

## Pre-publication history

The pre-publication history for this paper can be accessed here:

http://www.biomedcentral.com/1471-2350/11/69/prepub

## Supplementary Material

Additional file 1 **Supplementary material**. Figure S1a LD relationship (r^2^) between the investigated *TNF*/*LTA *tag SNPs in the case-control dataset; Figure S1b LD relationship (r^2^) between the investigated *TNF*/*LTA *tag SNPs in the parent-offspring dataset; Table S1 Genotype counts of the 10 *TNF*/*LTA *tag SNPs passing quality control in the case-control dataset; Table S2 Genotype counts of 11 *TNF*/*LTA *tag SNPs in the T2D probands and their parents; Table S3 Power calculations based on each *TNF*/*LTA *tag SNPs Click here for file
