# Supplementary material for: Large-scale association analysis of TNF/LTA gene region polymorphisms in type 2 diabetes
Source: BMC Med Genet. 2010 May 6;11:69. doi: 10.1186/1471-2350-11-69 (PMC2873325; doi:10.1186/1471-2350-11-69)
Supplement: Additional file 1 — Supplementary material. Figure S1a LD relationship (r2) between the investigated TNF/LTA tag SNPs in the case-control dataset; Figure S1b LD relationship (r2) between the investigated TNF/LTA tag SNPs in the parent-offspring dataset; Table S1 Genotype counts of the 10 TNF/LTA tag SNPs passing quality control in the case-control dataset; Table S2 Genotype counts of 11 TNF/LTA tag SNPs in the T2D probands and their parents; Table S3 Power calculations based on each TNF/LTA tag SNPs [file 1471-2350-11-69-S1.DOC]

**SUPPLEMENTARY MATERIAL**


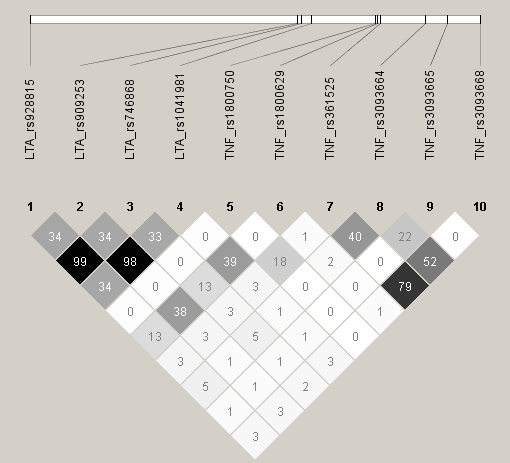


**Figure S1a**. LD relationship (r^2^) between the investigated *TNF*/*LTA* tag SNPs in the case-control dataset


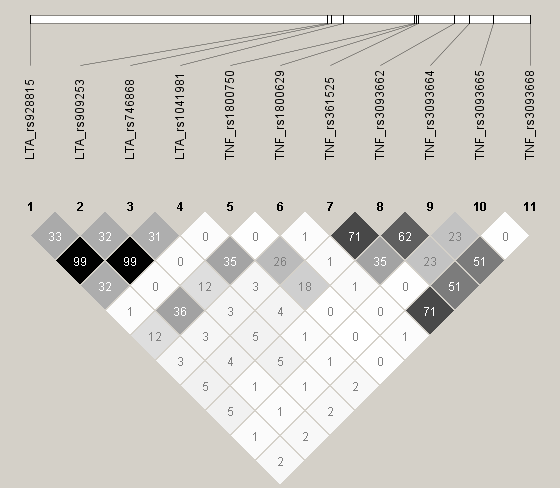


**Figure S1b**. LD relationship (r^2^) between the investigated *TNF*/*LTA* tag SNPs in the parent-offspring dataset

**Table S1**. Genotype counts of the 10 *TNF*/*LTA* tag SNPs passing quality control in the case-control dataset.

| SNP | Minor allele (1) | Major  allele (2) | N controls | 11/12/22  controls | N  cases | 11/12/22  cases |
| --- | --- | --- | --- | --- | --- | --- |
| rs928815 | T | G | 2493 | 353/1122/1018 | 1488 | 215/699/574 |
| rs909253 | C | T | 2500 | 377/1152/971 | 1485 | 182/687/616 |
| rs746868 | C | G | 2527 | 355/1128/1044 | 1495 | 214/704/577 |
| rs1041981 (T60N) | A | C | 2360 | 355/1078/927 | 1458 | 180/667/611 |
| rs1800750 | A | G | 2539 | 0/61/2478 | 1497 | 0/36/1461 |
| rs1800629 (G-308A) | G | A | 2504 | 97/774/1633 | 1454 | 39/477/938 |
| rs361525 (G-238A) | A | G | 2518 | 6/288/2224 | 1504 | 3/170/1331 |
| rs3093664 | G | A | 2518 | 15/416/2087 | 1485 | 11/240/1234 |
| rs3093665 | C | A | 2522 | 1/96/2425 | 1495 | 1/67/1427 |
| rs3093668 | C | G | 2536 | 3/236/2297 | 1496 | 3/144/1349 |

**Table S2**. Genotype counts of 11 *TNF*/*LTA* tag SNPs in the T2D probands and their parents.

| SNP | Minor | Major | 11/12/22 | 11/12/22 |
| --- | --- | --- | --- | --- |
|  | allele (1) | allele (2) | Parents | T2D probands |
| rs928815 | T | G | 111/350/307 | 48/179/155 |
| rs909253 | C | T | 95/363/313 | 44/183/151 |
| rs746868 | C | G | 112/347/308 | 48/182/154 |
| rs1041981 (T60N) | A | C | 93/351/307 | 45/178/154 |
| rs1800750 | A | G | 0/25/746 | 0/9/374 |
| rs1800629 (G-308A) | G | A | 32/201/451 | 11/111/223 |
| rs361525 (G-238A) | A | G | 2/88/679 | 0/42/340 |
| rs3093662 | G | A | 5/112/648 | 0/61/324 |
| rs3093664 | G | A | 3/116/649 | 1/63/316 |
| rs3093665 | C | A | 0/30/735 | 0/18/367 |
| rs3093668 | C | G | 0/67/701 | 0/34/348 |

**Table S3**. Power calculations based on each *TNF*/*LTA* tag SNPs

a) Number of cases required to detect 80% power at α=0.05 for a range of effect sizes under a log-additive model of inheritance.

| SNP | MAF | N_contr | N_case | C/C ^a^ | OR_MA | OR_1.1 | OR_1.2 | OR_1.3 | OR_1.4 | OR_1.5 | OR_1.6 | OR_1.7 | OR_1.8 | OR_1.9 | OR_2 |
| --- | --- | --- | --- | --- | --- | --- | --- | --- | --- | --- | --- | --- | --- | --- | --- |
| rs928815 | 0.379 | 2493 | 1488 | 1.675 | 1.056 | 2898 | 785 | 377 | 228 | 157 | 117 | 91 | 75 | 63 | 54 |
| rs909253 | 0.354 | 2500 | 1485 | 1.683 | 0.889 (1.125) | 2970 | 803 | 385 | 232 | 159 | 118 | 93 | 75 | 63 | 54 |
| rs746868 | 0.378 | 2527 | 1495 | 1.69 | 1.066 | 2891 | 783 | 376 | 228 | 156 | 116 | 91 | 74 | 62 | 54 |
| rs1041981 | 0.352 | 2360 | 1458 | 1.619 | 0.892 (1.121) | 3021 | 817 | 391 | 236 | 162 | 120 | 94 | 77 | 64 | 55 |
| rs1800750 | 0.012 | 2539 | 1497 | 1.696 | 1.001 | 55073 | 14374 | 6655 | 3891 | 2584 | 1860 | 1413 | 1118 | 912 | 761 |
| rs1800629 | 0.191 | 2504 | 1454 | 1.722 | 0.984 (1.016) | 4283 | 1139 | 537 | 320 | 216 | 158 | 122 | 98 | 82 | 69 |
| rs361525 | 0.058 | 2518 | 1504 | 1.674 | 0.981 (1.019) | 12070 | 3166 | 1473 | 866 | 578 | 418 | 319 | 254 | 208 | 174 |
| rs3093664 | 0.088 | 2518 | 1485 | 1.696 | 0.996 (1.004) | 8203 | 2158 | 1007 | 594 | 397 | 288 | 221 | 176 | 145 | 122 |
| rs3093665 | 0.023 | 2522 | 1495 | 1.687 | 1.192 | 29151 | 7618 | 3531 | 2067 | 1375 | 990 | 754 | 597 | 487 | 407 |
| rs3093668 | 0.050 | 2536 | 1496 | 1.695 | 1.053 | 13806 | 3618 | 1682 | 987 | 658 | 476 | 363 | 288 | 236 | 198 |

^a^C/C = number of controls/number of cases. The required number of controls is C/CxN (cases); MA = minor allele

b) Number of parent-offspring trios required to detect 80% power at α=0.05 for a range of effect sizes under a log-additive model of inheritance.

| SNP | MAF | N_FamTrio | OR_MA ^a^ | OR_1.1 | OR_1.2 | OR_1.3 | OR_1.4 | OR_1.5 | OR_1.6 | OR_1.7 | OR_1.8 | OR_1.9 | OR_2 |
| --- | --- | --- | --- | --- | --- | --- | --- | --- | --- | --- | --- | --- | --- |
| rs928815 | 0.359 | 322 | 0.8 (1.25) | 3708 | 1004 | 481 | 291 | 200 | 148 | 116 | 95 | 79 | 68 |
| rs909253 | 0.358 | 321 | 1.083 | 3713 | 1005 | 482 | 291 | 200 | 148 | 116 | 95 | 80 | 68 |
| rs746868 | 0.362 | 324 | 0.828 (1.208) | 3696 | 1001 | 480 | 290 | 199 | 148 | 116 | 95 | 79 | 68 |
| rs1041981 | 0.355 | 301 | 1.058 | 3726 | 1008 | 483 | 292 | 200 | 149 | 117 | 95 | 80 | 68 |
| rs1800750 | 0.012 | 327 | 0.583 (1.715) | 69569 | 18224 | 8465 | 4966 | 3308 | 2386 | 1818 | 1442 | 1178 | 986 |
| rs1800629 | 0.195 | 237 | 1.043 | 5350 | 1427 | 674 | 402 | 273 | 200 | 155 | 125 | 103 | 88 |
| rs361525 | 0.055 | 324 | 1 | 15938 | 4193 | 1956 | 1152 | 771 | 558 | 427 | 340 | 279 | 234 |
| rs3093662 | 0.079 | 321 | 1.068 | 11412 | 3009 | 1407 | 831 | 557 | 404 | 310 | 247 | 203 | 171 |
| rs3093664 | 0.086 | 321 | 1.279 | 10571 | 2789 | 1305 | 771 | 517 | 376 | 288 | 230 | 189 | 160 |
| rs3093665 | 0.023 | 324 | 1.167 | 36746 | 9636 | 4481 | 2631 | 1755 | 1267 | 967 | 767 | 628 | 526 |
| rs3093668 | 0.045 | 321 | 1.409 | 19256 | 5061 | 2358 | 1388 | 927 | 671 | 513 | 408 | 335 | 281 |

^a^MA = minor allele
